# Role of epithelial integrin-linked kinase in promoting intestinal inflammation: effects on CCL2, fibronectin and the T cell repertoire

**DOI:** 10.1186/1471-2172-12-42

**Published:** 2011-08-01

**Authors:** Kiran Assi, Scott Patterson, Shoukat Dedhar, David Owen, Megan Levings, Baljinder Salh

**Affiliations:** 1 Department of Medicine, The Jack Bell Research Centre, 2660 Oak Street, Vancouver, British Columbia, V6H 3Z6, Canada; 2 Department of Surgery, The Jack Bell Research Centre, 2660 Oak Street, Vancouver, British Columbia,V6H 3Z6, Canada; 3 British Columbia Cancer Research Centre, 10-110 675 West 10th Avenue, Vancouver, British Columbia, V5Z 1L3, Canada; 4 Department of Anatomical Pathology, The Jack Bell Research Centre, 2660 Oak Street, Vancouver, British Columbia, V6H 3Z6, Canada

**Keywords:** ILK, colitis, CCL2, fibronectin, T regulatory cells

## Abstract

**Background:**

The role of integrin signaling in mucosal inflammation is presently unknown. Hence, we aimed to investigate the role of epithelial-derived integrin-linked kinase (ILK), a critical integrin signaling intermediary molecule, in colonic inflammation.

**Methods:**

Conditional intestinal epithelial cell ILK knockout mice were used for assessment of acute and chronic dextran sodium sulfate (DSS) -induced colitis. Disease activity was scored using standard histological scoring, mucosal cytokines were measured using ELISA, chemokines were determined using reverse-transcription polymerase chain reaction, as well as Q-PCR, and intracellular cytokine staining performed using FACS analysis.

**Results:**

In both acute and chronic DSS-induced colitis, compared to wild-type mice, ILK-ko mice exhibit less weight loss, and have reduced inflammatory scores. In an *in vitro *model system using HCT116 cells, we demonstrate that si-RNA mediated down-regulation of ILK results in a reduction in monocyte chemoattractant protein 1 (MCP1, CCL2) chemokine expression. A reduction in CCL2 levels is also observed in the tissue lysates of chronically inflamed colons from ILK-ko mice. Examination of mesenteric lymph node lymphocytes from ILK-ko mice reveals that there is a reduction in the levels of IFN gamma using intracellular staining, together with an increase in Foxp3+ T regulatory cells. Immunohistochemistry demonstrates that reduced fibronectin expression characterizes the inflammatory lesions within the colons of ILK-ko mice. Intriguingly, we demonstrate that fibronectin is directly capable of downregulating T regulatory cell development.

**Conclusions:**

Collectively, the data indicate for the first time that ILK plays a pro-inflammatory role in intestinal inflammation, through effects on chemokine expression, the extracellular matrix and immune tolerance.

## Background

Inflammatory bowel diseases are chronic disorders that commonly affect individuals in the second to third decades of life. They are relatively common in the northern hemisphere, and are also being increasingly recognized in the developing world. A number of different genetic mutations are associated with these diseases, and typically result in a dysregulated immune response to the bacteria residing within the host gut [1,2]. The inflammatory response in IBD is characterized by an influx of lymphocytes, monocytes and polymorphonuclear leukocytes, which can all mediate chronic tissue damage. Tumor necrosis factor alpha (TNFα) appears to be a key factor in this process, as neutralization of this critical cytokine is highly efficacious in treating both Crohn's disease and ulcerative colitis [3,4].

Integrin-linked kinase (ILK) is an adapter protein that links the extracellular matrix with the cell interior through its interactions with the cytoplasmic tails of certain integrins. ILK plays a critical role in development, as its knockdown results in failure of implantation of the trophoblast. When ILK is deleted in specific tissues, processes such as cardiac development; angiogenesis; cartilage growth; skin growth; gut development and T-cell migration can all be impaired [5]. ILK also has an important role in cancer since it has oncogenic properties when overexpressed in cancer cell lines, and high ILK expression in a variety of malignancies is associated with a negative prognosis [6]. There is controversy surrounding its ability to regulate the activity of protein kinase B/Akt, but recent findings indicate that ILK acts in concert with other molecules such as Pinch and Rictor to achieve this effect [7,8].

There is scant data as yet on the role of ILK in inflammation. Although T cell-specific deletion of ILK did not establish a role in T cell proliferation, impaired migration was described [9]. We have previously observed that mice with a conditional epithelial cell knockout of ILK develop smaller tumors, in response to chronic inflammation associated with exposure to azoxymethane and dextran sodium sulfate administration [10] suggesting that ILK may regulate inflammation. In order to investigate this hypothesis we investigated the role of epithelial cell-specific expression of ILK in acute and chronic models of colitis. We report that ILK-ko mice are significantly protected from colitis; protection from disease is associated with altered expression of the chemokine, CCL2, and fibronectin. Moreover, ILK-ko mice have increased numbers of mucosal Tregs, consistent with the finding that fibronectin can directly impact on T regulatory cell development *in vitro*.

## Methods

### Materials

Anti-FoxP3 antibody was obtained from eBiosciences (San Diego, CA); anti-fibronectin antibody from Abcam (Cambridge, MA); anti-alpha 5 integrin antibody, alpha 5 integrin si-RNA, and ILK antibody from Santa Cruz (Santa Cruz, CA); anti-CD3, anti-CD28 antibody from BD Biosciences. ELISA kits for TNFα, IFNγ, IL-12p40, CCL2 were obtained from BD Biosciences (Mississauga, ON). QLT0267 was kindly provided by QLT Inc, Vancouver, BC, Canada. Horse-radish peroxidase conjugated secondary antibodies were obtained through Calbiochem (San Diego, CA). EGTA, EDTA, MOPS, PMSF, sodium orthovanadate, leupeptin, aprotinin, benzamidine, dithiothreitol and β-glycerolphosphate, were purchased from Sigma.

### Animal handling, processing, colitis induction and analysis

Mice on an FVB (Friend virus B-type) background were used for all the experimental work in this project. All animals were kept in conventional housing in the animal care facility at Jack Bell Research Centre. They were fed chow ad libitum and had liberal access to drinking water. All experiments were approved by the UBC Animal Ethics Committee (A05-1580). Colonic epithelial cell specific inactivation of ILK was achieved by crossing the Fabp -Cre mice with the ILK^flox/flox ^animals. The resulting offspring were then backcrossed with the homozygote floxed mice to generate the genotype: ILK^flox/flox^,Cre. Genotyping for Cre and ILK were carried out as previously described [10]. Briefly, tail DNA was obtained and the following primers used to detect Cre expression: 5'-CCTGGAAAATGCTTCTGTCCG-3' and 5'-CAGGGTGTTATAACAATCCC-3'. ILK genotype was monitored using: 5'-CCAGGTGGCAGAGGTAAGTA-3' and 5'-CAAGAAATAAGGTGAGCTTCAGAA-3'.

Acute and chronic colitis were induced using previously well-described methods [11] in 10 week old mice of mixed gender. In the acute colitis model mice were given 3.5% DSS in their drinking water for 1 week and then terminated after 1 more week (ie at 12 weeks). In the chronic colitis model, mice were started on the first of three cycles of 5 days of 2.5% DSS given in the drinking water followed by 1 week of normal tap water. Mice were monitored daily for illness behaviour, weight recording and sacrificed on day 36 (ie at just over 15 weeks of age). Their colons were examined for macroscopic and H& E for microscopic disease activity as previously described [12]. After removal, the colons were fixed in 10% buffered formalin for immunohistochemistry, protein lysates were prepared for western analysis and ELISA. Mesenteric lymph nodes and spleens were removed, and lymphocytes harvested for intracellular cytokine staining for FoxP3 and IFNγ as described below.

### SDS-Polyacrylamide Gel Electrophoresis

Colonic tissue or cultured cells were homogenized in buffer containing 20 mM MOPS, 150 mM NaCl, 50 mM β-glycerophosphate, 5 mM EGTA, 50 mM NaF, 1 mM DTT, 1 mM sodium vanadate, 0.5% NP40 and 1 mM PMSF. After sonicating for 15 s (x 2) and centrifuging at 14,000 RPM for 15 min, the protein concentration in the supernatant was determined by the Bradford assay (Bio-Rad, Mississauga, Ont). 25 ug of protein from each sample was resolved using 10% SDS-PAGE before transferring to nitrocellulose membranes (Bio-Rad). The blots were blocked in 5% skim milk in TBST (20 mM Tris-HCl pH 7.4, 250 mM NaCl, 0.05% Tween-20) for 1 h before probing for 2 h using the appropriate primary antibody. The blots were washed with TBST for 10 min three times, before being incubated with the appropriate secondary antibody for 1 h. Following 3 further washes in TBST, they were developed using the enhanced chemiluminescence detection system (ECL, Amersham, Montreal, Quebec).

### Immunohistochemistry

Paraffin-embedded colonic tissue samples were de-waxed in xylene twice for 5 min, rehydrated in a series of ethanol (100% - 70%) for 3 min each followed by rehydration in PBS for 30 min. After rehydration the endogenous peroxidase was blocked with 0.3% hydrogen peroxide followed by antigen retrieval by microwaving sections in citrate buffer pH 6.0 (10 mM Na citrate). Following antigen retrieval, the sections were washed three times with PBS, blocked in 1% BSA for 1 h, and then stained using the Vectastain ABC kit (Vector laboratories, Burlingame, CA) mentioned below according to manufacturer's recommendations but with the following modifications. Sections were incubated with the following primary antibodies at 4°C overnight: ILK (1:100, Cell Signaling, Pickering, Ontario), fibronectin (1:200, Abcam, Cambridge, MA), anti-Foxp3 (1:100, eBiosciences, San Diego, CA), anti-CD3 (1:100, Dako Canada Inc., Mississauga, ON). Following incubation, the sections were rinsed three consecutive times with PBS and then incubated with the appropriate biotinylated secondary antibody for 1 h followed by incubation with peroxidase-labelled streptavidin. Nova -red and DAB were used as the chromagens, and the sections were counterstained with haematoxylin. Three blinded observers independently examined all stained sections.

For detection of Th17 cells by immunofluorescence, the slides were processed as for IHC and the following antibodies were used: DAPI and IL-17A (eBio 17B7). Sections were stained with Vectastain ABC elite kit and biotinylated ant-rabbit for DAPI, or eFluor650 Nanocrystal conjugation kit, cat no. 88-7072-98 antibody, and Avidin D-FITC (or Avidin-Texas Red) used for immunofluorescence (Vector laboratories, CA, USA). Each section had its own control using the secondary antibody only. Pre-immune serum was initially used to ensure specificity of the signal with each of the antibodies.

### Q-PCR

1 ug of RNA, obtained using Trizol from HCT116 cells, was reverse transcribed using random hexamers (Perkin-Elmer Applied Biosystems, Branchburg, NJ) and 20 units of Moloney murine leukemia virus reverse transcriptase M-MLV (Invitrogene) in 20 μl of total volume at 25°C for 10 min and at 37°C for 60 minutes. The resulting first-strand complementary DNA (cDNA) was used as template for the real-time quantitative-PCR. The Applied Biosystems 5700 Sequence Detection System (Perkin-Elmer Applied Biosystems, Foster City, CA) was used for real time monitoring of PCR amplification of cDNA using the SYBRO Green Universal PCR Master Mix protocol. Amplification of the following cDNAs was performed using the primers listed:

CCL2: F: CTCTGCCGCCCTTCTGTG; R: TGCATCTGGCTGAGCGAG

CXCL-8: F: GGCACAAACTTTCAGAGACAG; R: ACACAGAGCTGCAGAAATCAGG

IκBα: F: TTGGGTGCTGATGTCAATGC; R: AGGTCCACTGCGAGGTGAAG

Relative quantification of gene expression was performed using Beta-Actin as a control. Beta-Actin cDNA was amplified separately on a duplicate set of samples using standard primers from AB Applied Biosystems. The comparative Ct (cycle threshold) method was used for relative quantification of gene of interest mRNA.

Statistical significance was determined by ANOVA model. The C_T _value is defined as the cycle number in which the detected fluorescence exceeds the threshold value.

Where C_T_1(gene of interest) and C_T_1(Beta-Actin) represent the C_T _values for the treated samples, respectively. C_T_2(gene of interest) and C_T_2(Beta-Actin) represent the C_T _values for the untreated samples, respectively.

### Cell Culture

HCT 116 cells were a kind gift of Bert Vogelstein (Johns Hopkins, Baltimore, Maryland) and were cultured in McCoys 5A Medium (Gibco, Burlington, Ontario) containing 10% heat inactivated fetal bovine serum (FBS) (Hyclone, Logan, Utah). Protein lysates were obtained using homogenization buffer as described above.

### Semiquantitative RT-PCR

1 ug of RNA, obtained using Trizol from HCT 116 cells (or murine colon), was reverse transcribed using random hexamers (Perkin-Elmer Applied Biosystems, Branchburg, NJ) and 20 units of Moloney murine leukemia virus reverse transcriptase M-MLV (Invitrogene) in 20 μl of total volume at 25°C for 10 min and at 37°C for 60 min. The resulting first-strand complementary DNA (cDNA) was used as template for the semi-quantitative-PCR. Amplification of the following cDNAs was performed using the primers listed: ILK(F): TTTTCACTCAGTGCCGGGAGG; (R): GTCCCTTGGCTTTGTCCACAG; CCL2 (F):ATGCAGGTCCCTGTCATGCTTCTG; (R):CTAGTTCACTGTCACACTGGTCACTCC;β-actin(F):AGAGGGAAATCGTGCGTGAC; (R):CAATAGTGATGACCTGGCGGT. Relative quantification of gene expression was performed using densitometry and beta-actin as a control.

### Si-RNA-mediated knockdown of ILK

This was performed as described previously [13] using a 21-mer to transfect HCT116 cells, grown to 60% confluency, using Silentfect (Biorad). Two separate ILK si-RNA and control (scrambled) sequences were purchased from Qiagen Inc (Mississauga, ON), and from Santa Cruz Biotechnology Corporation Inc (Santa Cruz, Ca). Gene knockdown was confirmed using western blotting and Q-PCR.

### Determination of regulatory T cell numbers and intracellular cytokine staining

To quantify Tregs and IFN-γ production ex vivo, mesenteric lymph nodes were collected, and stimulated with with phorbol myristate acetate (25 ng/ml) and ionomycin (1 mg/ml) for 6 h in the presence of brefeldin A (10 mg/ml) during the final 4 h. Cells were fixed and permeabilised using FOXP3-specific kit reagents (e-biosciences) and stained with anti-IFN-γ-PECy-7 (XMG1.2), anti-TNF-α-PE (JES5-16E3) or anti-IL-17-APC (17B7) (e-Biosciences). All samples were read on a BD FACS Canto and analyzed with FCS Express V3 (De Novo Software). To assess Treg development in vitro, T cells were purified and cultured in SFEM medium (StemCell Technologies), supplemented with 10 mM HEPES, 2 mM glutamine, 1 mM sodium pyruvate, 1 mM MEM non-essential amino acid solution and 100 U/ml each of Penicillin G and streptomycin. Flat-bottom plates were coated with aCD3 (10 mg/ml, 2C11) and serial dilutions of the indicated concentration of fibronectin (Sigma). Tregs were differentiated from CD4^+^CD25^- ^T cells in the absence or presence of soluble CD28 (1 mg/ml, 37.51), rhIL-2 (100 U/ml, Chiron) and rhTGF-β (10 ng/ml, R&D systems) as indicated.

### Statistical analysis

All macroscopic and histological disease scores, as well as cytokine levels were expressed as mean + SD, with p < 0.05 being considered significant using the Student's t-test (unpaired, two-tailed). Where indicated ANOVA was performed with Tukey post-hoc testing.

## Results

### Epithelial cell specific expression of ILK is induced by pro-inflammatory stimuli in a PI3-kinase and stress-activated protein kinase-dependent manner

**Figure 1:**
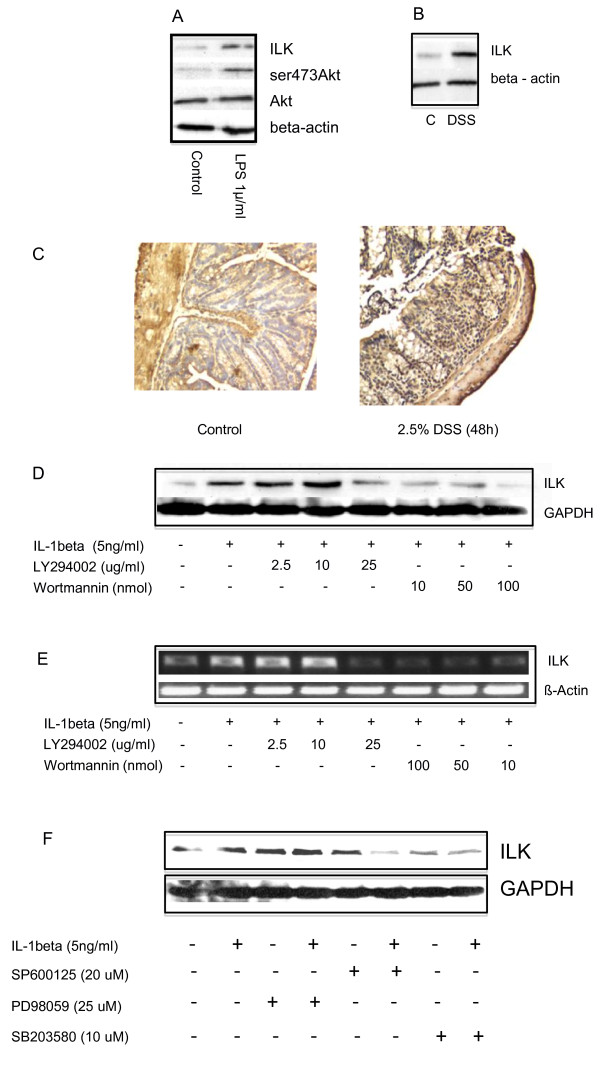
**ILK is induced by pro-inflammatory stimuli**. A. Colonic SW480 cells were stimulated with 1 ug/ml LPS for 24 h, and after harvesting the cells, western immunoblotting was performed. Membranes were probed with antibodies for ILK, ser473Akt, Akt and beta-actin. ILK protein induction is accompanied by an increase in the phosphorylation of Akt at ser473, but not the protein level of this kinase. The beta-actin is shown as an internal control. B. This shows the response of a murine colonic explant to exposure with 2.5% DSS for 48 h. There is an increase in the expression of ILK. The lower panel shows equivalent levels of beta-actin. C. Colons of control and DSS treated mice (48 h) were probed with a polyclonal antibody to ILK. Besides an increase in the intensity of the signal generally, more than 50% of the epithelial cell nuclei stain positively for ILK. D and E. Colonic HCT116 cells were stimulated with 5 ng/ml IL-1β for 4 h. Protein was obtained by lysing cells in homogenization buffer and western blotting performed for ILK expression (D). Alternatively, RNA was obtained by Trizol and used for cDNA synthesis; semi-quantitative RT-PCR for ILK was then performed using the primers indicated in materials and methods. Wortmannin and Ly294002 were both capable of inhibiting the IL-1β-induced ILK protein and mRNA. The β-actin signal is shown as the internal control. F. The experiment was repeated as in D with the addition of inhibitors of the MAPK pathway. Specifically PD98059, SP600125 and SB203580 were used to inhibit the p42/44 ERK, P54/45 JNK and p38SAPKs respectively, and GAPDH used as the loading control. Data are representative of 3 independent experiments for each panel.

We first used an in vitro system to determine whether ILK expression is modulated by inflammatory stimuli. SW480 colonic cells were exposed to LPS, and after 24 h ILK protein was induced, along with an increase in phosphorylation of Akt at ser473. Expression of Akt itself did not change (Figure 1A). To confirm these findings *in vivo*, colonic explants were exposed to DSS and as seen in Figure 1B, this also led to an increase in expression ILK protein expression. Histological examination of tissue sections demonstrated that increased ILK expression occurred both in the cytoplasm and nuclei of the epithelial cell compartment (Figure 1C). ILK expression was regulated by phosphatidylinositol 3-kinase, as both Ly294006 and wortmannin were capable of downregulating its induction at the protein level when HCT116 cells were stimulated with interleukin 1 beta (IL-1β, Figure 1D). Regulation of its induction occurs transcriptionally, as the same inhibitors attenuated the induction of ILK mRNA (Figure 1E). In order to determine whether other pathways were also implicated, we observed that both of the stress-activated protein kinase inhibitors SP600125 and SB203580 were also capable of a similar downregulation (Figure 1F).

### ILK regulates weight loss and inflammation in acute DSS-induced colitis

**Figure 2:**
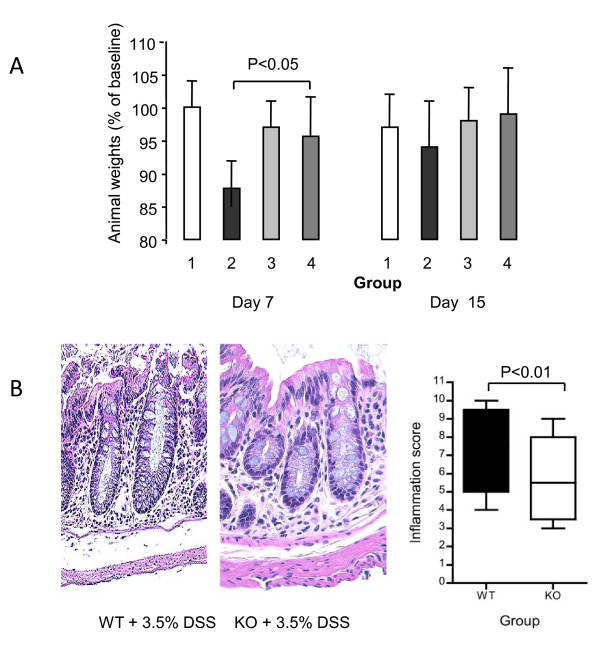
**Epithelial ILK provides a pro-inflammatory stimulus in acute colitis**. A. Acute colitis was induced in mice using 3.5% DSS in the drinking water. Mice were weighed daily, and the results after 7 days of DSS treatment, and following a further 8 days off DSS are shown. The weights expressed as the percentage of original (mean ± SEM) are shown (1 = WT control, 2 = WT+DSS, 3 = ILK-ko (C), 4 = ILK-ko + DSS). There is a significant attenuation of the normally observed weight loss in the ILK-ko mice at day 7 (p < 0.05, ANOVA, Tukey post-test). B. Representative histological slides for each of the DSS treated groups are shown. The inflammatory infiltrate, surface epithelial disruption and edema are reduced in the ILK-ko animals. There was a significant reduction in the inflammatory scores in the ILK-ko mice (n = 6 per group).

Evidence that ILK is highly expressed in inflammation at mucosal surfaces suggests it may be important in modulating gut immunity. This notion is supported by our previous observations in the colitis-associated cancer model [10], where ILK-ko mice had reduced inflammation-induced tumors. Hence we initially examined whether ILK-ko mice differed from their littermate controls in a model of acute colitis. Wild type and ILK-ko mice were treated with 3.5% DSS and at the end of 7 days (Figure 2A) there was a clear difference in the degree of weight loss observed in the ILK-ko mice as compared with the wild-type group. By the end of the second week these mice have recovered from the acute insult and hence the difference is no longer apparent. Histological examination confirmed the weight loss data since there was a significant attenuation of the inflammatory response in the ILK-ko mice (Figure 2B).

### ILK regulates the capacity of epithelial cells to produce CCL2 in vitro

**Figure 3:**
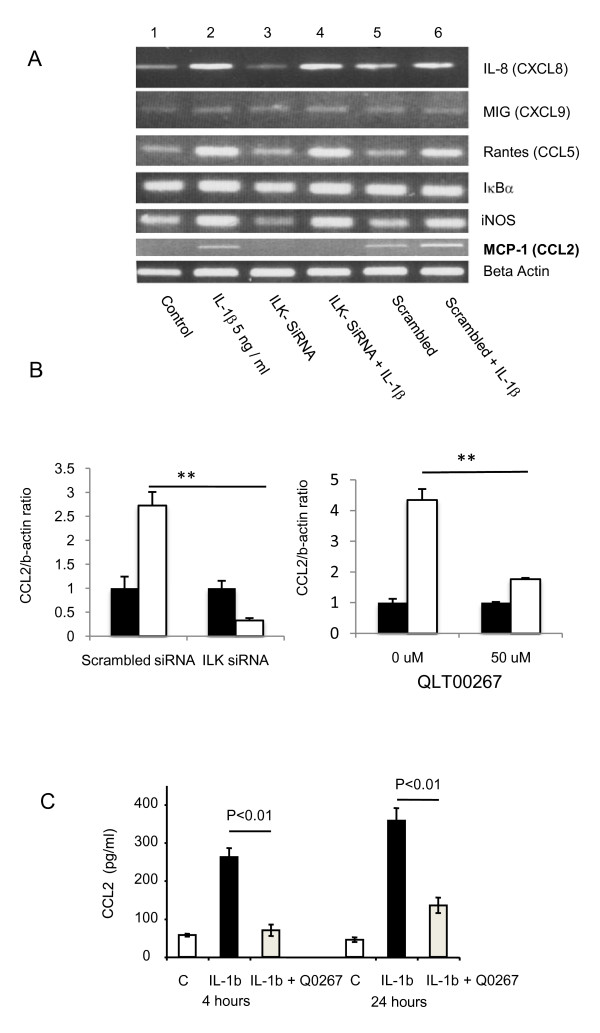
**ILK regulates CCL2 expression**. A. Si-RNA was used to knockdown ILK and a scrambled oligonucleotide was used as a control. RNA was extracted from HCT116 cells after 4 h of stimulation with IL-1β and reverse transcribed to make cDNA. This was used to determine the message of the molecules shown using RT-PCR. CCL2 induction in response to the cytokine IL-1β was the only molecule whose expression was blocked by si-RNA knockdown of ILK (see CCL2 panel, lane 4). This result was reproduced on 2 further occasions. B. The experiment was repeated as in A except Q-PCR used to determine the expression of CCL2, either following si-RNA knockdown of ILK (left panel) or using the QLT0267 inhibitor (right panel). C. HCT116 cells were pretreated with the specific ILK inhibitor QLT0267 for 1 hour before being stimulated with IL-1β for the times indicated. CCL2 concentrations were determined in the culture supernatent using ELISA. The data are representative of two separate experiments, each performed in triplicate (*p < 0.01).

Because of the reduced inflammatory cell infiltrate in the ILK-ko mice, we postulated that ILK regulates the ability of epithelial cells to express pro-inflammatory mediators. Hence we investigated the ability of si-RNA mediated knockdown of ILK to affect the expression of inflammatory-cytokine induced production of chemokines. As the data in Figure 3 indicate, exposure of HCT116 cells to IL-1β induces expression of IL-8 (CXCL8), Rantes (CCL5) and MCP1 (CCL2), but not MIG (CXCL9). Predictably, IL-1β also induces both IκBα and i-NOS. ILK knockdown had no effect on either IL-1β-induced CXCL8 or CCL5 expression but significantly inhibited the expression of CCL2. These data were confirmed not only by si-RNA to knockdown ILK and performing Q-PCR for CCL2, but also using a specific inhibitor of ILK signaling, QLT0267 [14], both of which resulted in reduced expression of CCL2 message (Figure 3B). We also investigated another unrelated si-RNA to knockdown ILK with similar effects on CCL2 expression (data not shown). ELISA further corroborated these results using the ILK inhibitor to detect IL-1β generated CCL2 protein. After a 4 h or 24 h exposure to QLT0267 there was a significant (> 70%) reduction in IL-1β-induced CCL2. These findings indicate that ILK is potentially capable of regulating epithelial cell function by modulating the expression of a well-described immune cell chemoattractant.

### Expression of ILK in epithelial cells influences chronic gut inflammation and production of CCL2 in vivo

**Figure 4:**
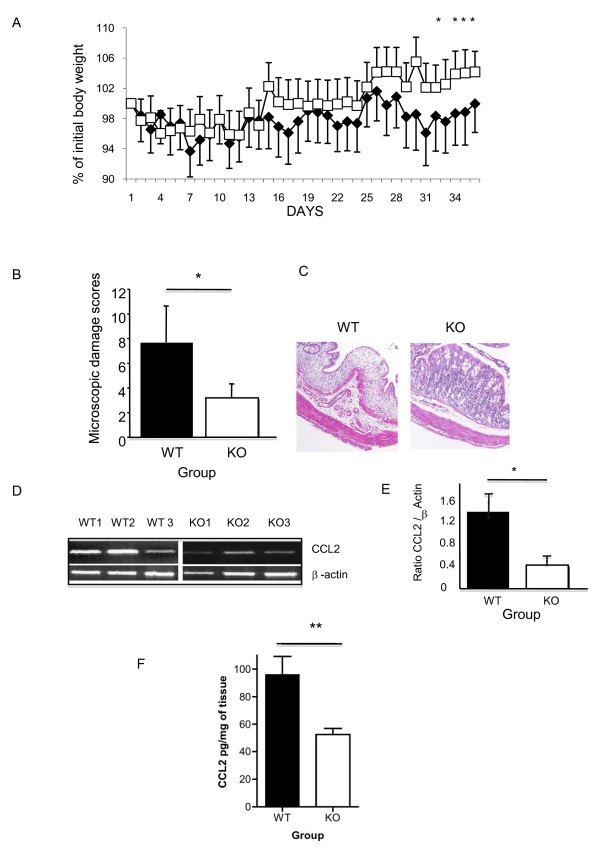
**ILK regulates wasting disease and CCL2 expression in chronic colitis**. A. Chronic DSS (2.5%) colitis was induced in 6 animals per group using 3 rounds of 2.5% DSS in the drinking water. Animal weights were monitored and the data in A show that there was a significant attenuation of the weight loss in the ILK-ko animals (squares), compared with the wild-type group (diamonds), (*p < 0.05). B. Assessment of microscopic damage in these mice indicated a significant reduction in the ILK-ko mice (*p < 0.05). Representative histology is shown in C, where an obvious loss of crypts, mucosal edema and inflammatory infiltrate are shown in the control sample. D. Total RNA was extracted from sections of distal colon and reverse transcribed to make cDNA. This was used to determine the message levels of CCL2 using semi-quantitative RT-PCR. As can be seen from this figure, CCL2 induction was significantly (*p < 0.05) reduced in the ILK-ko animals (the barchart E shows the message levels corrected for β-actin, and are for the entire sets of 6 mice per treatment group). F. Distal colonic lysates were obtained using homogenization buffer and then used to determine CCL2 levels by ELISA. The data are for 6 mice per group and show a reduction in levels of CCL2 in the ILK-ko mice (**p < 0.01).

We next investigated the impact of loss of ILK in epithelial cells in a model of chronic colitis, as this is considered to be more representative of human IBD. In this model, mice were treated with 3 cycles of 2.5% DSS for 5 days followed by 7 days without DSS treatment. As the weight chart indicates (Figure 4A), with each successive round of DSS there is a notable increasing separation between ILK-ko mice and wild-type mice. Specifically, after 36 days the amount of weight loss in the ILK-ko mice was significantly less than that of their wild-type counterparts. When the animals were sacrificed we observed reduced macroscopic disease scores in the ILK-ko group. These data were confirmed upon examination of histological sections where ILK-ko mice had significantly reduced inflammation and mucosal damage (Figure 4B, C). Based on our finding that ILK regulates expression of CCL2, we also measured expression of CCL2 mRNA and confirmed significantly reduced expression in the ILK-ko mice (Figure 4D, E). Tissue homogenates examined for protein levels of CCL2 further confirmed these data (Figure 4F). Collectively these data indicate that ILK normally promotes intestinal inflammation, and that ILK-mediated regulation of CCL2 production by epithelial cells may be involved in this response.

### Interconnection between ILK and fibronectin

**Figure 5:**
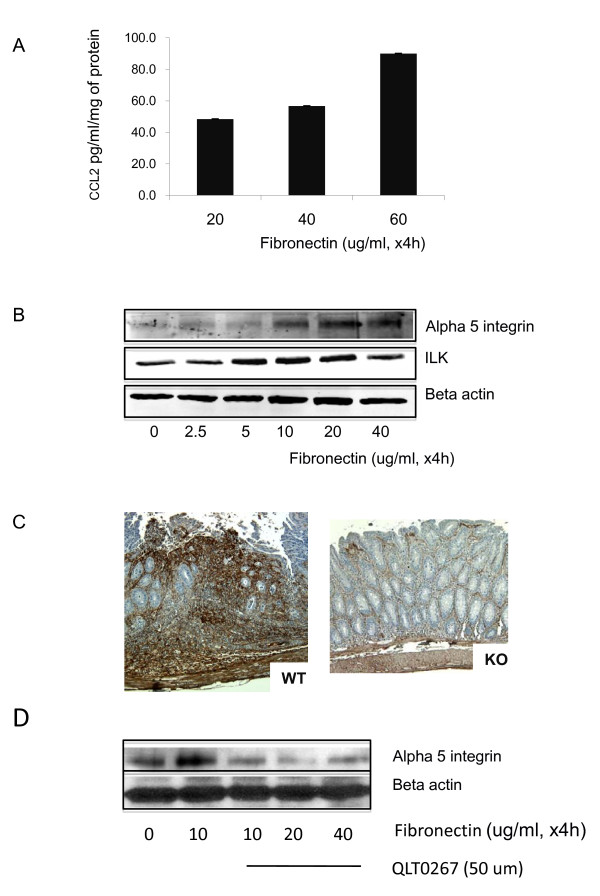
**Fibronectin regulates CCL2, ILK and α5 integrin**. HCT116 cells were plated on tissue culture plates coated with increasing levels of fibronectin for 4 h. In A, the CCL2 levels were measured in the medium using ELISA. In B, the cells were subsequently lysed and equivalent amounts of protein resolved using SDS page. After transfer the membranes were probed with the antibodies indicated. The data indicate increased ILK and α5 integrin expression with increasing fibronectin exposure, peaking at 20 ug/ml. C. Immunohistochemistry was performed on colonic sections using a fibronectin antibody on 3 different mice in each group. There is a clear reduction in fibronectin expression in the ILK knockout mice (a representative slide is shown). D. HCT116 cells were exposed to the doses of fibronectin indicated and the QLT 0267 compound added at the start of the experiment. The cell lysates were then resolved using SDS-PAGE and probed with either the α5 integrin or beta actin (loading control) antibodies. The data are representative of 3 separate experiments.

CCL2 is a chemokine with a role in mediating fibrosis in several systems, including the colon [15]. Intriguingly, one of the interesting facets of ILK function is its capacity to affect modulation of the extracellular matrix component, fibronectin [16]. Since fibronectin is associated with colitis and its expression levels undergo biphasic modulation during induction of inflammation and during healing [17], we speculated that loss of ILK in epithelial cells may also have an impact on this protein. We initially asked whether fibronectin is capable of regulating CCL2 expression by cultured epithelial cells. By plating cells on tissue culture plates coated with increasing levels of fibronectin we observed that there was an increase in the amount of CCL2 detected in the medium by ELISA (Figure 5A). We also wanted to determine whether fibronectin regulates the expression of its receptor (α5) and ILK. Using the same *in vitro *system we found that fibronectin stimulated a dose-dependent increase in expression of ILK and α5, peaking at 20 ug/ml (Figure 5B). Next, using immunohistochemistry we observed that there is an impressive reduction in fibronectin expression in the ILK-ko mice in comparison with the wild-type mice (Figure 5C). We also determined that QLT0267 was capable of preventing the fibronectin-mediated expression of α5 integrin (Figure 5D). Collectively, these data indicate the existence of a bidirectional pathway whereby an ILK-dependent mechanism is capable of regulating fibronectin expression levels in the ECM, which is itself capable of regulation ILK and its receptor α5 integrin, as well as CCL2, by epithelial cells.

### Expression of ILK in epithelial cells affects the infiltrating T cell profile

**Figure 6:**
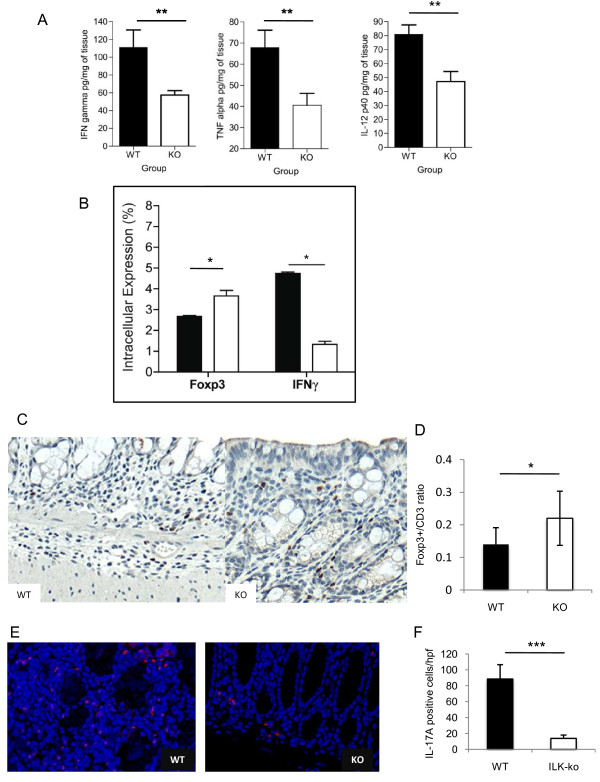
**Epithelial ILK regulates tissue expression of inflammatory cytokines**. A. Interferon gamma, tumor necrosis factor alpha and interleukin-12p40 cytokine levels were determined in colonic homogenates from 6 ILK-knockout animals and 6 wild-type controls (**p < 0.01). B. Lymphocytes were obtained from mesenteric lymph nodes of wild-type and ILK-ko mice. Intracellular staining for FoxP3 and IFNγ was performed as described in materials and methods. After stimulation with PMA (25 ng/ml) and ionomycin (1 mg/ml) for 6 h, cells were fixed and permeabilized. Then they were stained with the indicated antibodies and read on a BD FACS Canto. The data are from 6 ILK-ko and 6 wild-type mice (*p < 0.05). C. Tissue sections were obtained from control and ILK-ko mice at the end of 3 rounds of DSS treatment, and processed for immunohistochemistry. Using anti-CD3 and anti-FoxP3 antibodies, the number of positively staining cells were counted in 3 fields from 6 separate animals, in each group. The ratios obtained are shown in D (*p < 0.05). E. IL-17A staining was performed using immunofluorescence as described in methods for tissue sections from the same sets of mice as in C. The red staining cells are clearly observed to be more numerous in the control samples, and the data is graphically represented in F.

We next investigated whether the development of T cell responses was altered in ILK-ko mice. First, we analyzed production of pro-inflammatory cytokines in the colonic homogenates of the chronic DSS-induced colitis mice, and found that ILK-ko mice had significant reductions (up to 50%) in their levels of TNF-α, IFN-γ and IL-12p40 (Figure 6A). To specifically address the cytokine profiles within the T cell compartment, mesenteric lymph nodes were collected and intracellular staining was performed on CD4+ T cells. As shown in Figure 6B, the data indicate a significant reduction in the intracellular staining for IFNγ, in ILK-ko mice (Figure 6B, right panel), confirming an attenuated Th1 response.

Foxp3^+ ^Tregs are critical regulators of the intestinal immunity [18]. Based on the reduction in IFN-γ-producing T cells, were hypothesized that there may be a corresponding increase in Tregs. Indeed we found that the proportion of Tregs was significantly increased in mesenteric lymph nodes (Figure 6B, left panel). Based on these ex vivo results, we next used immunohistochemistry to examine the ratio of FoxP3 positive cells to total CD3 positive cells in mice affected with chronic colitis. These data confirmed that ILK-ko mice have a proportionately increased number of Tregs infiltrating their intestinal mucosa (Figure 6C, D). To determine the effect on Th17 cells, which are also critical determinants of colonic inflammation, immunofluorescence was performed. As the data indicate (Figure 6E and 6F) there is a significant reduction in the numbers of IL-17A positive T cells within the ILK-ko mice.

### Fibronectin is capable of regulating the development of T regulatory cells

**Figure 7:**
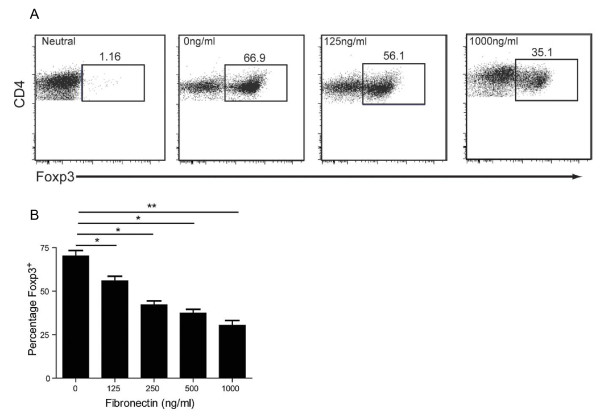
**Fibronectin regulates Treg differentiation**. T lymphocytes (CD4+CD25-) were obtained from the mesenteric lymph nodes of mice and exposed to plate bound anti-CD3 (10 mg/ml) and fibronectin, as well as soluble anti-CD28 (1 mg/ml), rIL-2 (100 u/ml) and TGFβ (10 ng/ml) as described in materials and methods. After 24 h the proportion of FoxP3+ cells were determined using FACS analysis. In A, i: representative neutral, 1000 ng/ml fibronectin; ii: iTreg, 0 ng/ml fibronectin; iii: iTreg, 125 ng/ml fibronectin; iv: iTreg, 1000 ng/ml fibronectin. The barchart in B represents the overall dose-response relationship, performed in triplicate and repeated twice. (*p < 0.05, **p < 0.01, using ANOVA).

Based on our findings that ILK-ko mice have reduced fibronectin and an increase in mucosal Tregs, and recent evidence that Treg function can be regulated by components of the extracellular matrix [19], we speculated that these two observations may be linked. To test whether the levels of fibronectin may directly affect Treg development, we isolated CD4^+^CD25^- ^T cells and stimulated them under Treg-inducing conditions (TGF-β and IL-2) in the absence or presence of increasing amounts of fibronectin. Remarkably, we found that fibronectin directly inhibits the development of Foxp3+ Tregs in a dose-dependent manner (Figure 7). These data not only reveal an inverse link between the levels of a major ECM component, fibronectin, and the differentiation of Tregs, but they also provide a possible mechanistic basis for the resistance of ILK-ko mice to colitis.

## Discussion

This work shows for the first time that the epithelial expression of ILK, a molecule with a critical role in bidirectional cellular signaling, impacts significantly on mucosal immunity. ILK-ko mice consistently exhibited less wasting disease in response to DSS-induced colitis, had reduced macroscopic and histological scores of disease, and reduced pro-inflammatory cytokine production. Not only was there a reduction in the levels of colonic tissue cytokines in the ILK-ko mice, but also a consistent reduction in the numbers of IFNγ staining cells in the MLN (and splenic) lymphocytes. These data demonstrate that ILK normally functions to promote pro-inflammatory effects on epithelial cells, at least in part, via stimulating expression of CCL2 and fibronectin expression, the latter of which acts on T cells to suppress the development of Tregs. Knowledge that Treg development is directly regulated by ECM proteins, provides a new paradigm in mucosal immunity and offers mechanistic insight into why ILK-ko mice are resistant to colitis.

We found that ILK regulates the expression of the chemokine CCL2, both *in vitro *using an epithelial cell line and possibly *in vivo *in colitic mice. This is important not only because this chemokine is upregulated in human IBD, with increased CCL2 levels having been reported in the mucosa of IBD patients as determined by immunohistochemistry and ELISA [20] but also, because mice with either this chemokine genetically deleted [21], or with its receptor (CCR2) deleted [22] are protected from developing experimental colitis. The net result of reduced CCL2 expression would be a reduction in the influx of monocytes and lymphocytes, leading to reduced inflammation in comparison to wild-type mice. Of especial interest is the recent description of an MCP-1 (CCL2) polymorphism that is associated with Crohn's disease [23], which may have implications for disease pathogenesis.

Fibronectin is a large (250 kDa) molecular weight glycoprotein present in various tissue compartments, with defined roles in cell adhesion, migration and proliferation. It exists as a dimer with each monomer constructed of repeating type I, II and III protein domains. Alternative splicing at extra domains A and B as well as at a connecting segment III leads to the occurrence of over 20 different forms [24]. Previously it has been shown that there is enhanced expression of fibronectin in inflamed ulcerative colitis mucosa and in fibrotic Crohn's disease, but a reduced expression in inflamed Crohn's disease mucosa [25]. Interestingly, it was virtually absent in intestinal fistulae from the latter population [26]. It is known that ILK regulates the epithelial expression of fibronectin, which is an important component of the extracellular matrix, both by means of over-expression and also gene knockdown studies [16,27]. Also, epithelial fibronectin is known to increase during both the acute and healing phases of colitis [17]. We have added to this by demonstrating that reduced inflammation in the ILK-ko mice is attended by a reduction in levels of fibronectin expression. Because fibronectin may mediate leucocyte binding [28], as these cells traverse the extracellular matrix, part of the explanation for reduced inflammation is simply that there are fewer lymphocytes present to mediate tissue damage. As many different cells are able to synthesize fibronectin we can conclude that at least a part of this is due to its reduced epithelial expression in ILK-ko mice. Our observation that ILK, CCL2 and α5 integrin are induced in response to fibronectin exposure, indicates an important connection between these molecules, possibly through a positive feedback loop.

Reconciling observations in chronic intestinal inflammation, where CCL2 is increased in both UC and CD, and changes in fibronectin expression, which appears to only undergo an increase in UC (and fibrotic Crohn's disease), is not straightforward. Furthermore, the DSS-induced colitis model utilized in this study is not one where fibrosis is generally recognized to occur. This suggests that another non-fibrosis associated, ILK-CCL2-fibronectin pathway exists in early inflammation, and that interference with any of these three components is capable of attenuating the inflammatory response. It is quite likely that alternative or additional mechanism(s) operate(s) to effect inflammation in Crohn's disease, and consequently changes in fibronectin expression are not observed. Future work will address the role of ILK on other components of the extracellular matrix that undergo changes in IBD.

Whilst changes in CCL2 and fibronectin may offer plausible explanations for the reduction in inflammation seen in the ILK-ko mice, we were keen to investigate potential additional immune mechanisms. The reduction in the level of gamma-interferon indicates a reduction in the Th1 response, and this observation was seen in tissue homogenates, as well as in CD4(+) cells harvested from the mesenteric lymph nodes. We next turned to T regulatory cells, which are known to have a fundamental role in regulation of mucosal immunity [18]. Indeed, we found that protection of ILK-ko mice from colitis correlated with a relative increase in Foxp3+ Tregs in both the colon and in mesenteric lymph nodes. Peripheral Treg development in the gut can be driven by many different tolerogenic signals such as TGF-β, retinoic acid and IL-10. We found that under the influence of TGF-β, fibronectin directly inhibited the development of Foxp3+ cells, for the first time providing a link between this and the development of immune tolerance. This finding complements recent data showing that another ECM protein, high molecular weight hyaluronan, the ligand for CD44, can promote the expression of FoxP3 [19], further supporting the notion that the integrity of the tissue matrix has a direct role in directing mucosal immune responses. Moreover, the direct effects of fibronectin on Treg development provide a mechanism by which the loss of ILK in epithelial cells could lead to a reduced susceptibility to colitis. Future research will be required to define how this novel aspect of Treg development is regulated at the molecular level.

There is considerable support for the involvement of another T cell population in the pathogenesis of IBD, specifically the Th17 cell [29]. In contrast to Th1 (requiring IFNγ, and IL-12), Th2 (requiring IL-4) and Treg (requiring TGFβ and IL-2) cells, this particular cell is dependent upon TGFβ, IL-6 and IL-23 for its differentiation [30]. It produces a different set of cytokines that include IL-17, IL-22 and TNFα, and is characterized by the expression of the transcription factor RORγt. An inverse relationship between a genetic polymorphism of its surface IL-23 receptor (IL-23R) and Crohn's disease has been described [31]. Recent work indicates some degree of T cell plasticity in that T cells that express both IL-17 and RORγt have been described [32]. Our work indicates that the increased FoxP3+ cell population in the ILK-ko mice is associated with a concomitant reduction in the IL-17+ cell population, in response to induction of chronic colitis. The presence of a smaller population of dual IL-17+/FoxP3+ cells cannot be excluded. Furthermore we do not observe any comparable change in the Th17 cell fraction (as compared to Tregs) upon plating onto fibronectin coated plates, indicating a specific effect for Treg generation.

## Conclusion

Our work provides the first striking example of an intestinal epithelial cell molecule ILK, capable of influencing the surrounding inflammatory milieu (through CCL2), as well as the ECM (through fibronectin), which in turn may impact on the mucosal inflammatory response (via Th1, Th17 and Treg cells). We therefore conclude that modulation of ILK signaling may have an impact on human IBD, and that this merits attention.

## Abbreviations

ILK: integrin-linked kinase; ILK-ko: ILK-intestinal epithelial cell knockout; CCL2: chemokine (C-C motif) ligand 2.

## Competing interests

The authors declare that they have no competing interests.

## Authors' contributions

KA and SP performed the experimental work; DO provided input into interpretation of pathology; SD provided essential reagents; ML provided input into experimental design and edited the manuscript; BS was responsible for conception and design, as well as writing the first and final versions of the manuscript. All authors have read and approve of the final version of the manuscript.
